# Supplementary material for: The interactome of intact mitochondria by cross-linking mass spectrometry provides evidence for coexisting respiratory supercomplexes
Source: Mol Cell Proteomics. 2017 Dec 8;17(2):216–32. doi: 10.1074/mcp.RA117.000470 (PMC5795388; doi:10.1074/mcp.RA117.000470)
Supplement: Supplemental Data [file supp_RA117.000470_133922_0_supp_23978_fzffwf.zip › spectra_annotation/mito_spectra_annotation/292-1-6-1-8-1.pdf]

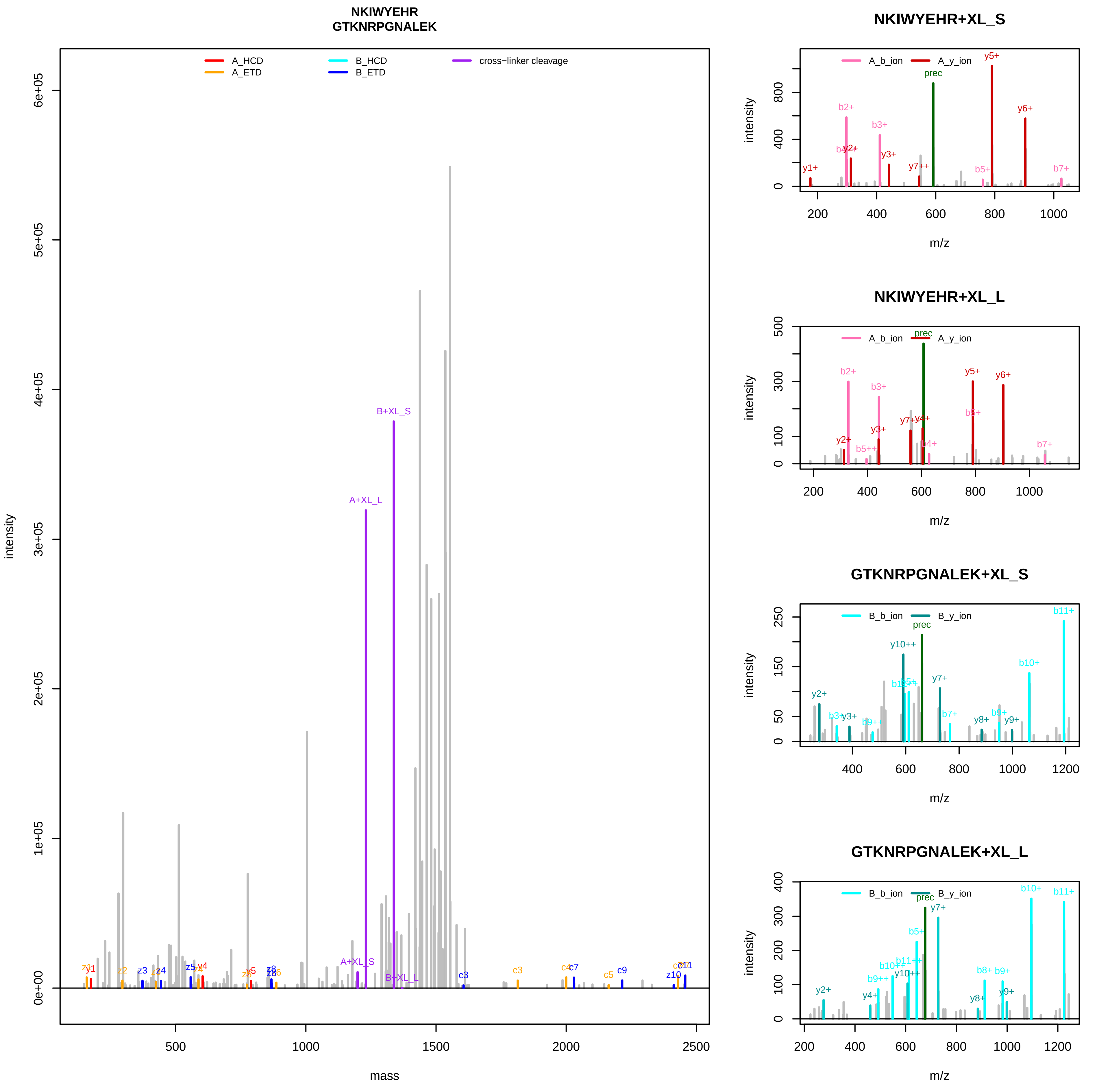
NKIWYEHR
GTKNRPGNALEK
A_HCD
A_ETD
B_HCD
B_ETD
cross−linker cleavage
B+XL_S
A+XL_L
A+XL_S
B+XL_L
intensity
mass
0e+00
1e+05
2e+05
3e+05
4e+05
5e+05
6e+05
500
1000
1500
2000
2500
y1
z2
z3
z4
z5
y4
y5
z8
c3
c4
c5
c7
c9
z10
c11
NKIWYEHR+XL_S
A_b_ion
A_y_ion
prec
y1+
y2+
y3+
y5+
y6+
y7++
b2+
b3+
b5+
b7+
m/z
NKIWYEHR+XL_L
b5++
GTKNRPGNALEK+XL_S
B_b_ion
B_y_ion
y2+
y3+
y7+
y8+
y9+
y10++
b3+
b7+
b9+
b10+
b11+
GTKNRPGNALEK+XL_L
y4+
b8+
b9++
b10++
b11++
y10++
b5+
